# Lipid Reorganization Induced by Shiga Toxin Clustering on Planar Membranes

**DOI:** 10.1371/journal.pone.0006238

**Published:** 2009-07-16

**Authors:** Barbara Windschiegl, Alexander Orth, Winfried Römer, Ludwig Berland, Bahne Stechmann, Patricia Bassereau, Ludger Johannes, Claudia Steinem

**Affiliations:** 1 Institut für Organische und Biomolekulare Chemie, Georg-August Universität, Göttingen, Germany; 2 Institut Curie, Centre de Recherche, CNRS UMR 144, Laboratoire Trafic, Signalisation et Ciblage Intracellulaires, Paris, France; 3 Institut Curie, Centre de Recherche, CNRS UMR 168, Laboratoire Physico-Chimie, Paris, France; The University of Manchester, United Kingdom

## Abstract

The homopentameric B-subunit of bacterial protein Shiga toxin (STxB) binds to the glycolipid Gb_3_ in plasma membranes, which is the initial step for entering cells by a clathrin-independent mechanism. It has been suggested that protein clustering and lipid reorganization determine toxin uptake into cells. Here, we elucidated the molecular requirements for STxB induced Gb_3_ clustering and for the proposed lipid reorganization in planar membranes. The influence of binding site III of the B-subunit as well as the Gb_3_ lipid structure was investigated by means of high resolution methods such as fluorescence and scanning force microscopy. STxB was found to form protein clusters on homogenous 1,2-dioleoyl-*sn*-glycero-3-phosphocholine (DOPC)/cholesterol/Gb_3_ (65∶30∶5) bilayers. In contrast, membranes composed of DOPC/cholesterol/sphingomyelin/Gb_3_ (40∶35∶20∶5) phase separate into a liquid ordered and liquid disordered phase. Dependent on the fatty acid composition of Gb_3_, STxB-Gb_3_ complexes organize within the liquid ordered phase upon protein binding. Our findings suggest that STxB is capable of forming a new membrane phase that is characterized by lipid compaction. The significance of this finding is discussed in the context of Shiga toxin-induced formation of endocytic membrane invaginations.

## Introduction

Shiga toxin (STx) produced by *Shigella dysenteriae* is a member of the so-called AB_5_ class of bacterial toxins. STx consists of a hexameric assembly comprising a single catalytically active A-subunit possessing rRNA-specific *N*-glycosidase activity and a B-subunit (STxB), a pentamer of identical fragments, which is responsible for the toxin binding to the plasma membrane of the host cell [1]. This homopentameric STxB can bind up to 15 molecules of the plasma membrane-embedded neutral receptor globotriaosylceramide (Gb_3_) [2], [3] and allows the intracellular transport of the holotoxin and the delivery of the monomeric A-subunit into the cytosol leading to the inhibition of protein biosynthesis [4].

Once bound to the plasma membrane, it has been shown that STx can be internalized in a clathrin- and caveolae-independent process [5]–[8], and a recent study demonstrates that the lipids themselves contribute significantly to the earliest steps of toxin uptake into cells, that is, the formation of membrane invaginations [9]. These membrane invaginations appear to be concomitant with membrane reorganization, as deduced from experiments using the polarization sensitive membrane dye Laurdan on cells [9]. In particular, STxB binds with high apparent affinity to Gb_3_ resulting in the formation of dynamic STxB clusters in a process that imposes negative curvature on the membrane [9], [10]. Coarse grained membrane simulations have demonstrated that curvature-mediated interactions can lead to protein clustering and the formation of invaginations [11]. In the case of STxB, the exact mechanism, by which local curvature is induced, still has not been described, even if it has been suggested, based on theoretical considerations that local lipid compaction might be the driving force [9].

Three different Gb_3_ binding sites (I, II, and III) are found on each of the five B-fragments that constitute one homopentameric STxB subunit, and it is known that they contribute differently to the binding of STxB to cells. While a mutation in binding site I (D17E-STxB) decreases the binding affinity to cells by a factor of about three [12], a mutation in binding site III (W34A-STxB) does not. However, despite the same binding affinity, the latter mutant shows a strongly reduced binding capacity suggesting that binding site III serves as a recognition element for additional Gb_3_ molecules after the protein has been bound [12]. The recruitment of Gb_3_ molecules underneath STxB via binding site III may then result in the formation of Gb_3_ clusters and membrane thickening as proposed by Ling et al. [3]. This, in turn, suggests that the molecular structure of Gb_3_, i.e. the composition of the fatty acids, which determines the localization of the lipids in phase separated lipid bilayers [13], [14] contributes considerably to Gb_3_ cluster formation. By molecular modeling, Nyholm et al. [15] observed an influence of the fatty acid composition on the conformation and position of the Gb_3_ head group at the membrane interface, which directly impacts the STxB binding affinity. This is further corroborated by the finding that the binding affinity of STxB to bilayers composed of phosphatidylcholine (PC) and Gb_3_ increases if the fatty acid chain length of PC decreases, which exposes the head group of Gb_3_ more into the aqueous phase [16]. Of note, the binding affinity of STxB to Gb_3_ was found to be larger if the receptor lipid was extracted from tissue, i.e. a mixture of Gb_3_ molecules was presented, compared to neat synthetic Gb_3_ molecules [17]. Furthermore, not only a higher binding affinity but also a larger binding capacity was found if a mixture of Gb_3_ lipids varying in the fatty acid composition was used for STxB binding studies, when compared to single Gb_3_ species [18]. These studies demonstrate a direct link between lipid structure and Shiga toxin interaction with cell membranes. The functional consequences of these differences have not yet been addressed directly.

In this study, we analyzed the molecular requirements for STxB-induced Gb_3_ clustering and for the previously suggested lipid reorganization in planar membranes. The influence of binding site III of the protein as well as of the Gb_3_ lipid structure on the lateral organization of STxB and the membrane was investigated by means of high resolution methods such as fluorescence and scanning force microscopy.

## Results

### Interaction of STxB with DOPC/cholesterol/Gb_3_ bilayers

**Figure 1:**
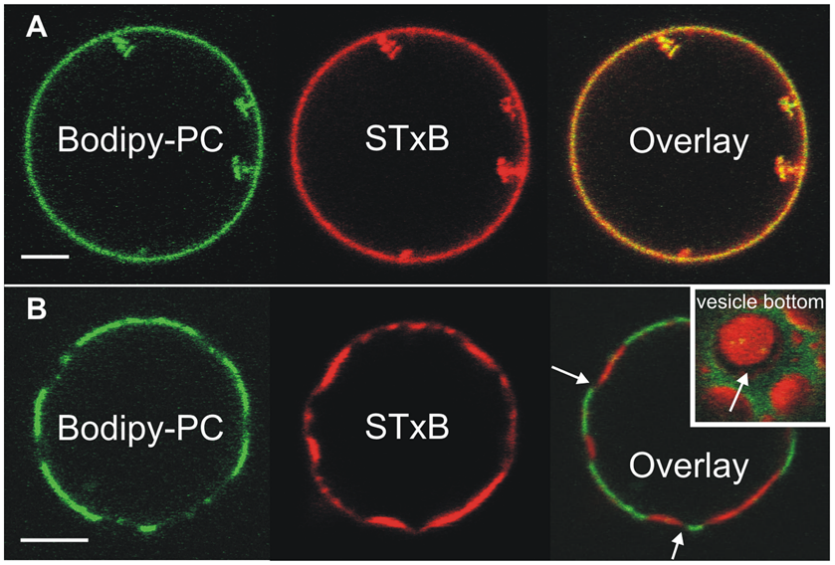
STxB binding to GUVs. (A) GUVs are composed of DOPC/cholesterol/porcine Gb_3_ (65∶30∶5) doped with 1 mol% Bodipy-PC. The formation of tubular membrane invaginations containing STxB (red) and membrane components (green) was observed. Scale bar: 5 µm. (B) GUVs are composed of DOPC/sphingomyelin/cholesterol/porcine Gb_3_ (41∶41∶13∶5) doped with 1 mol% Bodipy-PC. The protein (red) binds to already phase-separated GUV membranes (green). No tubule formation was observed but shallow invaginations. Scale bar: 5 µm.

To investigate the formation of membrane invaginations induced by STxB binding, first a lipid mixture composed of 1,2-dioleoyl-*sn*-glycero-3-phosphocholine (DOPC) and cholesterol was chosen, doped with 5 mol% of purified porcine globotriaosylceramide (porcine Gb_3_). The cholesterol content was adjusted to 30 mol% in accordance with its typical abundance in the plasma membrane of eukaryotic animal cells. The specific binding of Cy3-labeled STxB to DOPC/cholesterol/porcine Gb_3_ (65∶30∶5) giant unilamellar vesicles (GUVs, 10–30 µm in diameter) doped with 1 mol% Bodipy-PC at room temperature results in the formation of tubular membrane invaginations containing STxB (red fluorescence) and membrane components (green fluorescence) (Figure 1A) similar to what has been observed in experiments performed at 37°C [9].

**Figure 2:**
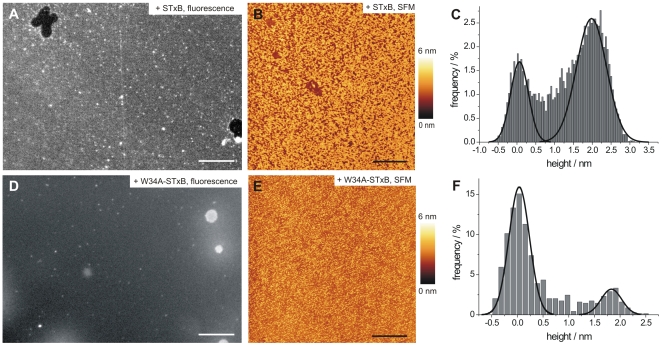
Binding of STxB to solid supported bilayers composed of DOPC/cholesterol/porcine Gb_3_ (65∶30∶5). (A) Fluorescence image after Cy3-labeled STxB incubation. Scale bar: 15 µm. (B) SFM image after protein binding shows homogeneously distributed STxB clusters. Scale bar: 1 µm. (C) The height difference between protein-free and STxB covered membrane is (2.2±0.4) nm (*n* = 35). (D) Fluorescence micrograph after incubation with 60 nM Cy3-labeled W34A-STxB. Scale bar: 15 µm. (E) The SFM image shows homogeneously distributed protein clusters. Scale bar: 2 µm. (F) The height difference between protein-free and protein-covered membrane is (1.6±0.3) nm (*n* = 12).

To elucidate protein- and lipid reorganization processes with high lateral resolution upon STxB binding, we made use of fluorescence and scanning force microscopy (SFM) on planar solid supported membranes (SSMs). As membrane bending is constrained in SSMs, the observed protein distribution is not influenced by membrane deformation. The topography of planar membranes composed of DOPC/cholesterol/porcine Gb_3_ (65∶30∶5) immobilized on atomically flat mica surfaces is featureless, i.e. no membrane domains are discernable (Figure S1), which is consistent with the homogeneous green Bodipy-PC fluorescence observed in GUVs (data not shown). In rare cases, small holes were identified as membrane defects confirming the formation of a bilayer on the surface. Binding of Cy3-labeled STxB results in a rather homogeneous fluorescence on the membrane, which is the result of high protein coverage of membrane bound STxB (Figure 2A). Only very rarely, black areas were found, which are assigned to membrane defects as proven by SFM (data not shown), while small white spots are attributed to larger protein aggregates attached to the surface. If the specific receptor Gb_3_ is not present in the membrane, very week fluorescence was detected as a result of non-specific protein binding, mainly localized in membrane defects.

By means of SFM, the lateral organization of membrane-bound protein was visualized showing micrometer-sized, sometimes interconnected STxB clusters (Figure 2B). A detailed analysis of the protein clusters reveals an overall protein coverage of about 30–60%, while the height difference between membrane and protein turned out to be (2.2±0.4) nm (*n* = 35, Figure 2C).

To elucidate the influence of binding site III of STxB on the distribution of the protein on the membrane, the binding behavior of a STxB variant, in which the Gb_3_ binding site III is inactivated by mutagenesis (W34A-STxB), was analyzed by fluorescence and scanning force microscopy. Even though the mutant protein still binds to membranes composed of DOPC/cholesterol/porcine Gb_3_ (65∶30∶5) as demonstrated by fluorescence microscopy (Figure 2D), the overall protein cluster size appears to be smaller (Figure 2E). As the molecular dimensions of the mutant protein are identical to those of the wild-type protein, the height difference between membrane and protein was expected to be the same. In contrast to this prediction, we found that the height difference between membrane and protein was significantly smaller, as a value of (1.6±0.3) nm (*n* = 12) was determined (Figure 2F). These results demonstrate how the protein's Gb_3_ binding capability modifies the overall membrane structure at STxB domains.

### Interaction of STxB with DOPC/sphingomyelin/cholesterol/Gb_3_ bilayers

In contrast to the lipid mixture discussed in the previous section, GUV membranes composed of a lipid raft mixture, namely DOPC/sphingomyelin/cholesterol/porcine Gb_3_ (41∶41∶13∶5) doped with 1 mol% Bodipy-PC was used. These membranes are phase separated at room temperature as concluded from the observed inhomogeneous Bodipy-PC fluorescence (data not shown). The fluorophore is excluded from the liquid ordered (*l*_o_-) phase, while it is well soluble in the liquid disordered (*l*_d_-) phase mainly composed of DOPC. The dark *l*_o_-phase is largely enriched in sphingomyelin, and cholesterol [19]–[21]. Cy3-labeled STxB is found at the *l*_o_-phase (Figure 1B) implying the preferential localization of porcine Gb_3_ in the *l*_o_-phase after protein binding. Interestingly, STxB fluorescence found at the border of the *l*_o_- and *l*_d_-phase is very faint, which might be a result of a preferential STxB clustering within the *l*_o_-domains (Figure 1B, overlay, vesicle bottom). Tubule formation was, however, not observed with this lipid composition after protein binding but only shallow invaginations, even after vesicle deflation.

**Figure 3:**
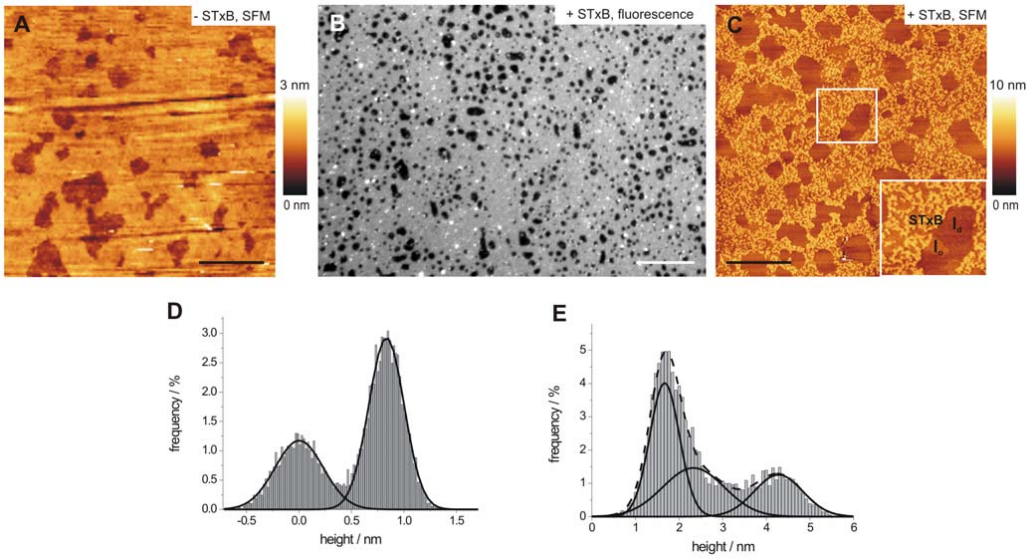
Binding of STxB to solid supported bilayers composed of DOPC/sphingomyelin/cholesterol/porcine Gb_3_ (40∶35∶20∶5). (A) In the absence of STxB the topographic SFM image of the bilayer shows the coexistence of a *l*_o_- and *l*_d_-phase. Scale bar: 5 µm. (B) Fluorescence micrograph after Cy3-labeled STxB incubation. Scale bar: 15 µm. (C) The SFM image shows STxB clusters located at the *l*_o_-phase. Scale bar: 5 µm. (D) Statistical height analysis reveals the *l*_o_-phase to be (0.7±0.2) nm (*n* = 46) higher than the *l*_d_-phase. (E) The height difference between STxB and the *l*_o_-phase exhibits (2.2±0.2) nm (*n* = 26) (typical histograms are shown, respectively).

To elucidate this observation in more detail, planar bilayers composed of DOPC/sphingomyelin/cholesterol/porcine Gb_3_ (40∶35∶20∶5) were analyzed by SFM, also showing the coexistence of two lipid phases (Figure 3A). The lower phase is assigned to the *l*_d_-phase enriched in DOPC, while the *l*_o_-phase exhibits a membrane thickness, which is (0.7±0.2) nm (*n* = 46) larger than the *l*_d_-phase as a result of a more ordered lipid packing of sphingomyelin, and cholesterol (Figure 3D), as reported previously [22]–[25].

Addition of Cy3-labeled STxB results in an inhomogeneous protein fluorescence (Figure 3B). A correlation of this fluorescence with SFM images allowed us to resolve the lateral protein distribution and membrane structure in more detail. Three different heights were found in the SFM topography image (Figure 3C). The lowermost structures are attributed to the *l*_d_-phase and correspond to the dark areas in the fluorescence image with respect to form, distribution and number. As no protein fluorescence is emitted from those areas, it is concluded that STxB does not bind to the *l*_d_-phase. The areas of bright protein fluorescence are a result of Cy3-labeled STxB bound to the liquid ordered phase (Figure 3B). By means of SFM imaging (Figure 3C) fractal STxB clusters bound to porcine Gb_3_, which are located in the *l*_o_-phase, became discernable. Only small areas in the *l*_o_-phase can be identified, on which no protein has been bound to, which might be explained by a local depletion of the receptor lipid Gb_3_. The height difference between the lowermost (*l*_d_-phase) and the observed medium height level (*l*_o_-phase without protein) was determined by line analysis, confirming the expected height difference of 0.6 nm between *l*_d_- and *l*_o_-phase prior to protein addition [22]–[25]. We could not observe any preferential binding of the protein to the interface region of *l*_o_- and *l*_d_-phase, which makes a clear distinction of these areas possible. From areas, where only the *l*_o_-phase and the protein submonolayer were present, we determined the height difference between adsorbed STxB and the *l*_o_-phase rather accurately to a value of (2.2±0.2) nm (*n* = 26) by histogram analysis (Figure S2). A histogram of an area that contains all three different heights (*l*_o_-phase, *l*_d_-phase, and protein layer) reproduces the obtained height differences, but it is more difficult to unambiguously separate them (Figure 3E).

### Lipid reorganization in planar bilayers by STxB binding

**Figure 4:**
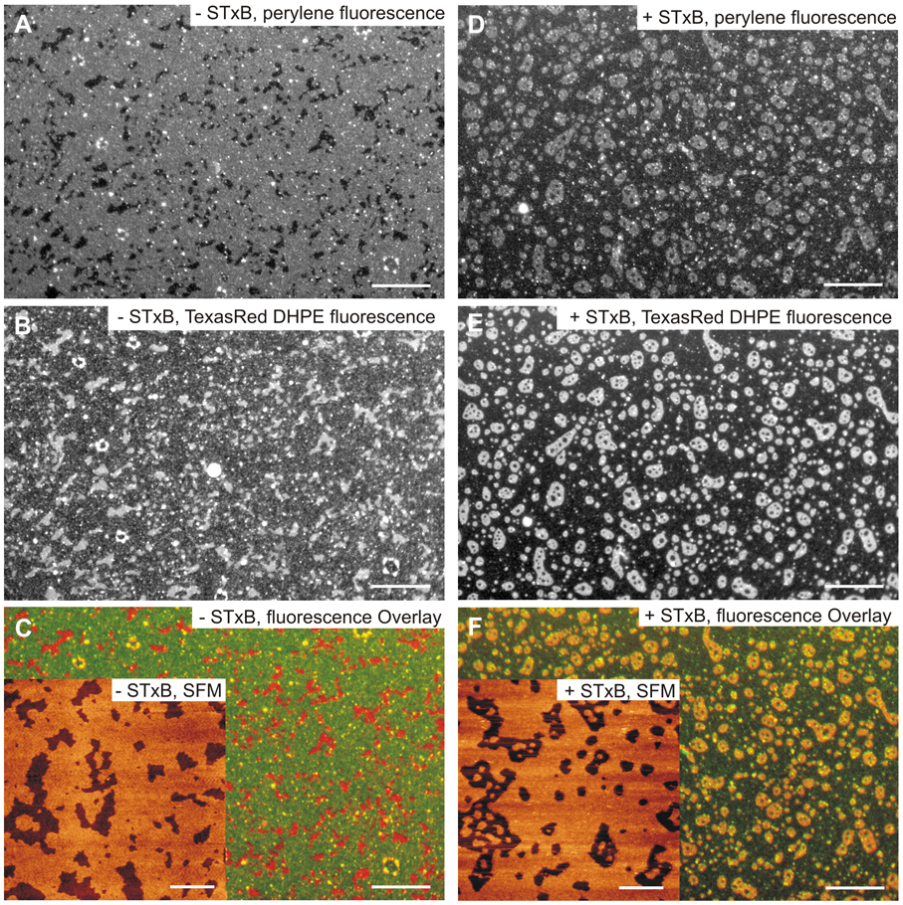
Fluorescence and scanning force micrographs of solid supported bilayers composed of DOPC/sphingomyelin/cholesterol/porcine Gb_3_ (40∶35∶20∶5). (A, B, C) Fluorescence images prior to the addition of STxB. (A) 0.2 mol% perylene replaces DOPC and its fluorescence visualizes the *l*_o_-phase. Scale bar: 20 µm. (B) The *l*_d_-phase of the same area was detected with the fluorophore TexasRed DHPE (0.1 mol%). Scale bar: 20 µm. (C) Overlay of the fluorescence images shown in (A) and (B) together with the corresponding SFM image (inset). The images are false colored with the TexasRed DHPE fluorescence in red and the perylene fluorescence in green. Scale bar: 20 µm. (D, E, F) Fluorescence images after incubation of the membrane with 60 nM STxB. (D) The fluorophore perylene is enriched in the *l*_d_-phase. Scale bar: 20 µm. (E) The fluorophore TexasRed DHPE is found in the same lipid phase, the *l*_d_-phase. Scale bar: 20 µm. (F) Overlay of the fluorescence images shown in (D) and (E). The SFM image shows small *l*_o_-domains within the *l*_d_-domains (F, inset). Scale bar: 20 µm.

It is assumed that STxB binding to the *l*_o_-phase of DOPC/sphingomyelin/cholesterol/porcine Gb_3_ (40∶35∶20∶5) membranes results in lipid reorganization. To prove this hypothesis, planar membranes were labeled with two different fluorescent lipids. 0.1 mol% of DOPC were replaced by TexasRed DHPE, which is preferentially localized in the *l*_d_-phase, while further 0.2 mol% DOPC were substituted by perylene. Figure 4A/C (green color) show perylene fluorescence images prior to the addition of STxB. Large fluorescently labeled areas are discernable, with dark domains in between, where the perylene fluorophore is excluded. The black areas in the perylene fluorescence appear bright in the TexasRed DHPE fluorescence images (Figure 4B/C, red color) confirming that the perylene fluorophore is mainly localized in the *l*_o_-phase. Small white spots are assigned to residual vesicles adsorbed on the bilayer.

**Figure 5:**
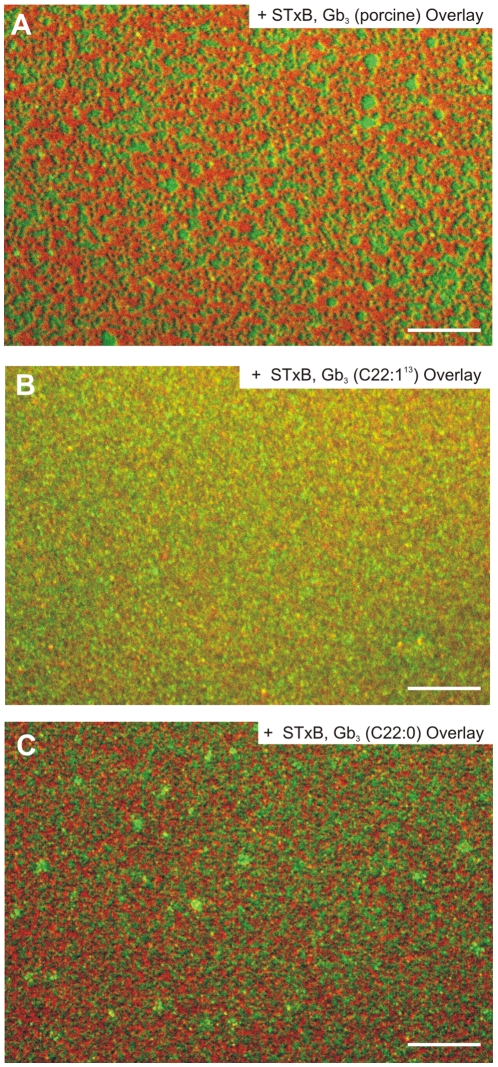
Overlay of fluorescence images of DOPC/sphingomyelin/cholesterol/Gb_3_ (40∶35∶20∶5) planar bilayers after STxB binding. The Cy3-labeled STxB is shown in red, the perylene fluorescence in green. (A) Porcine Gb_3_. Scale bar: 15 µm. (B) Gb_3_ (22∶1^13^). Scale bar: 15 µm. (C) Gb_3_ (22∶0). Scale bar: 15 µm.

After protein incubation, the appearance of the fluorescence images has changed considerably. Rather circular domains up to 10 µm in diameter are visible in the TexasRed DHPE fluorescence (Figure 4E/F, red color) assigning the *l*_d_-phase. To quantify this, the circularity of the *l*_d_-domains was determined, which increases from 0.66±0.21 to 0.81±0.17 after protein binding. The same circularity of the *l*_d_-domains (0.83±0.15) was found by analyzing the Cy3-fluorescence image after STxB binding (Figure 3B). Of note, also the perylene fluorophore is now preferentially located in the *l*_d_-phase (Figure 4D/F, green color) indicating that the partition of the perylene fluorophore between the *l*_o_- and *l*_d_-phase has changed upon STxB binding. This observation is further supported by an overlay image showing both, the fluorescence of Cy3-labeled STxB and perylene, which demonstrates that the protein is localized at the *l*_o_-phase, from which the perylene fluorophore is excluded (Figure 5A). We suggest that a tighter packing of the lipids in the *l*_o_-phase, as a result of the formation of STxB-Gb_3_ complexes, leads to the exclusion of the perylene fluorophore from this lipid phase. This tighter lipid packing in the *l*_o_-phase is also reflected in the fraction area of *l*_o_-phase in the bilayer, which decreases from 72% prior to protein binding to 63% after protein adsorption as determined by pixel analysis of the fluorescence images (see Figure 4B/E). Furthermore, SFM images (Figure 4C/F, insets) reveal that, after STxB has been bound, small *l*_o_-domains appear within the *l*_d_-domains, which could not be resolved by fluorescence microscopy.

As Gb_3_ appears to be the major player for lipid reorganization and compaction, we further elucidated the impact of the packing parameter of Gb_3_ on the redistribution of perylene, which indicates lipid compaction, by defining and varying its bound fatty acid side chain (Gb_3_ (22∶0) and Gb_3_ (22∶1^13^)). Compared to membranes doped with porcine Gb_3_ (Figure 5A), the protein appears rather homogeneously distributed on Gb_3_ (22∶1^13^) doped bilayers (Figure 5B), while an unambiguous separation of perylene and Cy3-labeled protein fluorescence is observed for Gb_3_ (22∶0) (Figure 5C) containing membranes. These results obtained on phase-separated planar supported membranes demonstrate the strong influence of the fatty acid side chain on the protein distribution and on cluster formation.

### STxB binding to lipid monolayer systems

**Figure 6:**
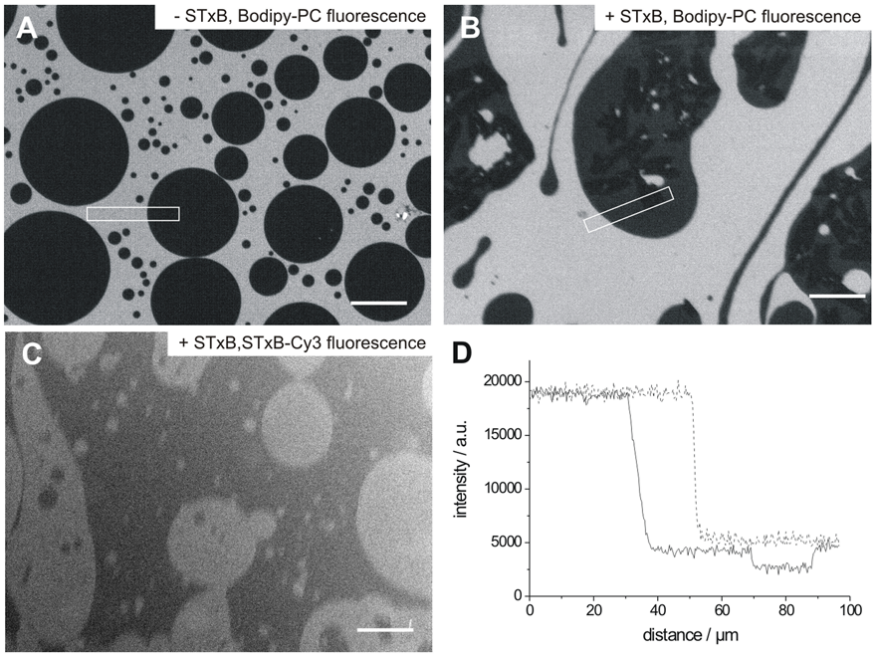
Fluorescence micrographs of lipid monolayers at the air/water interface. The monolayers are composed of DOPC doped with 1 mol% Bodipy-PC/sphingomyelin/cholesterol/porcine Gb_3_ in a molar ratio of 40∶35∶20∶5. (A) Without bound protein, round dark domains were observed at a surface pressure of 30 mN m^−1^, which are assigned to the *l*_o_-phase. Scale bar: 50 µm. (B) Bodipy-PC fluorescence after injecting 60 nM Cy3-labeled STxB into the PBS buffered subphase. Scale bar: 50 µm. (C) Cy3-STxB fluorescence after the addition of protein. Scale bar: 50 µm. (D) Average profiles of 40 line scans of the fluorescence intensity obtained in the white rectangles shown in Figure (A) (dotted line) and (B) (solid line).

By using planar lipid monolayers at the air/water interface, a versatile system is available, which enabled us to monitor changes in membrane domains and the formation of lipid phases at a defined surface pressure. A lipid mixture composed of DOPC doped with 1 mol% Bodipy-PC/sphingomyelin/cholesterol/porcine Gb_3_ (40∶35∶20∶5) was spread on a PBS buffered subphase, the monolayer compressed to a surface pressure of 30 mN m^−1^ (Figure S3) and the phase behavior analyzed by fluorescence microscopy. At 30 mN m^−1^, coexistence of two lipid phases was observed. Round as well as elongated domains were discernable, which are assigned to the *l*_o_-phase enriched in sphingomyelin, and cholesterol (Figure 6A) [26], [27]. Bodipy-PC is localized in the surrounding *l*_d_-phase mostly composed of DOPC. Cy3-labeled STxB was injected into the subphase, which enabled us to monitor the Bodipy-PC and Cy3-fluorescence quasi-simultaneously after the protein had been bound to the monolayer. After protein binding, the Bodipy-PC fluorescence images show rather irregularly shaped dark *l*_o_-phase domains, which appear bright in the Cy3-fluorescence indicating that the protein is solely adsorbed on the *l*_o_-phase domains (Figure 6C). A detailed pixel analysis of the Bodipy-PC fluorescence images reveals that the overall area occupied by *l*_o_-phase does not change considerably upon protein binding. However, 30 minutes after protein addition, the number of *l*_o_-phase domains per monolayer area has increased from (330±70) mm^−2^ to (750±290) mm^−2^, which is primarily a result of an increase in the number of small domains (<1000 µm^2^) from 72% to 94% of all *l*_o_-phase domains. This result suggests that STxB binding to the monolayer induces new *l*_o_-phase domains similar to what has been observed in bilayers (compare Figure 4C/F, insets).

Surprisingly, darker areas become discernable within the larger *l*_o_-phase domains (Figure 6B) after protein binding. The slight change in fluorescence intensity can be more easily visualized by the intensity profile depicted in Figure 6D. This finding implies lipid compaction within the existing *l*_o_-phase, leading to a strong exclusion of the Bodipy-PC fluorophore.

**Figure 7:**
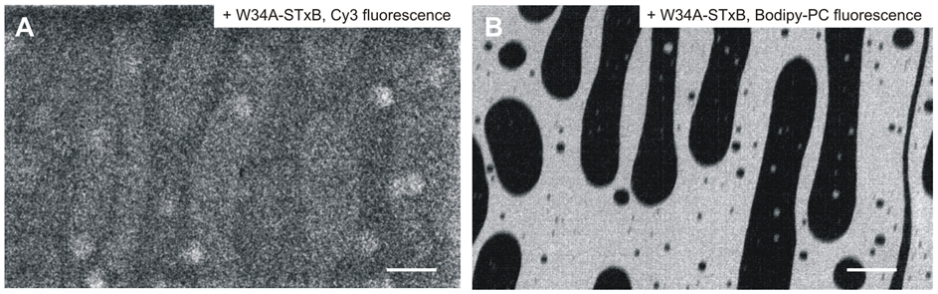
Fluorescence micrographs of lipid monolayers at the air/water interface. The monolayers are composed of DOPC/sphingomyelin/cholesterol/Gb_3_ in a molar ratio of 40∶35∶20∶5 doped with 1 mol% Bodipy-PC at a surface pressure of around 30 mN m^−1^. (A) Cy3-fluorescence image indicating binding of Cy3-labeled W34A-STxB to the *l*_o_-phase domains. Scale bar: 50 µm. (B) Corresponding Bodipy-PC fluorescence. Scale bar: 50 µm.

The same experiment was performed using the mutant W34A-STxB lacking binding site III. While Cy3-labeled protein binding to the *l*_o_-phase was observed (Figure 7A), the total number of domains per monolayer area decreased from (430±140) mm^−2^ prior protein addition to (320±120) mm^−2^ 30 minutes after STxB binding. Moreover, darker areas within the *l*_o_-phase were not found (Figure 7B).

**Figure 8:**
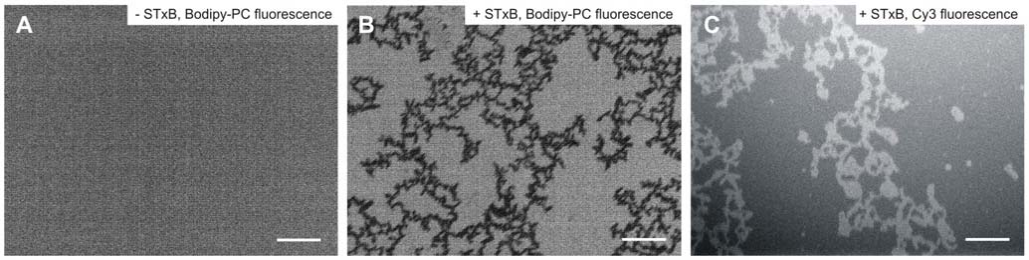
Fluorescence micrographs of lipid monolayers at the air/water interface. The monolayers are composed of DOPC doped with 1 mol% Bodipy-PC/sphingomyelin/cholesterol/porcine Gb_3_ in a molar ratio of 65∶10∶20∶5 at surface pressures between 25–30 mN m^−1^. (A) Bodipy-PC fluorescence image prior STxB addition. Scale bar: 50 µm. (B) After STxB addition small dark, ordered domains are formed. Scale bar: 50 µm. (C) Dark domains in the Bodipy-PC fluorescence appear bright in the Cy3-fluorescence. Scale bar: 50 µm.

To elucidate whether STxB is indeed capable of inducing new membrane domains, we started from a non-phase separated lipid monolayer and investigated, whether dark domains are induced upon binding of STxB. A monolayer composed of DOPC doped with 1 mol% Bodipy-PC/sphingomyelin/cholesterol/porcine Gb_3_ (65∶10∶20∶5) was spread at the air/water interface (Figure S3). Before protein binding, the monolayer does not show phase separation at a surface pressure of 10–35 mN m^−1^ as deduced from fluorescence images (Figure 8A). However, after STxB binding, dark domains with a dendritic shape become discernable in the Bodipy-PC fluorescence images (Figure 8B), which appear bright in the Cy3-fluorescence images demonstrating that the protein has been bound to these newly formed irregularly shaped liquid ordered domains (Figure 8C). These findings suggest that the protein is capable of forming *l*_o_-phase domains probably enriched in Gb_3_. The same experiment was performed with the mutant W34A-STxB and no formation of *l*_o_-phase domains was observed (data not shown).

## Discussion

Previously, it has been shown that Shiga toxin is able to form tubules and buds in cellular and artificial systems, which was discussed in terms of protein-lipid nanodomain formation [9]. Such domains might be a direct result of protein binding leading to receptor clustering [28], which, in turn, can modulate the entire membrane organization. Here, we have shown that tubule formation in GUVs occurs not only at 37°C but also at room temperature, if a DOPC/cholesterol/porcine Gb_3_ (65∶30∶5) lipid mixture is used. In contrast, only shallow deformations are formed in phase separated GUV membranes composed of DOPC/sphingomyelin/cholesterol/porcine Gb_3_ (41∶41∶13∶5) with an inhomogeneous Cy3-STxB fluorescence found at the border of the *l*_o_- and *l*_d_-phase suggesting STxB clustering within the *l*_o_-domains. In GUV experiments addressing cholera toxin binding to G_M1_ doped lipid bilayers, Baumgart et al. [20] were able to correlate the lipid domain composition and local membrane curvature proposing that these factors are important for membrane invagination processes.

Here, the combination of fluorescence and scanning force microscopy on membranes immobilized on solid substrates enabled us to investigate the organization of membrane bound STxB and elucidate the protein-induced reorganization of lipids within membranes. SFM images of STxB bound to DOPC/cholesterol/porcine Gb_3_ (65∶30∶5) and DOPC/sphingomyelin/cholesterol/porcine Gb_3_ (40∶35∶20∶5) bilayers revealed clustering of the protein similar to what has been observed for the lateral organization of cholera toxin bound to DOPC membranes containing G_M1_ [29]. Surprisingly, we found a mean height of the protein clusters of 2.2 nm, even though the protein height as deduced from the crystal structure of STxB is expected to be only 2 nm [30], [31]. In general, protein heights determined by SFM are often under-estimated [32] because of the force applied by the tip and a non-perfect protein packing. Indeed, membrane bound W34A-STxB with the same molecular dimensions, but only two Gb_3_ binding sites per monomer, exhibits a smaller height of 1.6 nm compared to that of the crystal structure as expected. Hence, we conclude that the observed 28% height increase of the protein domains compared to those of W34A-STxB is a result of membrane thickening induced by the accumulation of Gb_3_ owing to the occupation of binding site III of each STxB subunit after protein binding as proposed by Bast et al. [12] and Ling et al. [3]. A higher lipid packing density, as suggested by the perylene redistribution experiments (see below) in DOPC/sphingomyelin/cholesterol/Gb_3_ (40∶35∶20∶5) membranes (Figure 4 and 5) might also contribute to this bilayer thickening. This composition phase separates forming a *l*_o_- and *l*_d_-phase, which is in good agreement with reported ternary phase diagrams of DOPC, cholesterol and sphingomyelin [33]. STxB solely binds to Gb_3_ and is excluded from the *l*_d_-phase. Hence, it can be concluded that Gb_3_ is located within the *l*_o_-phase after STxB has been bound. For G_M1_, it has been reported that it is localized in the *l*_o_-phase of lipid monolayers composed of phosphatidylcholine, sphingomyelin and cholesterol [27], [34].

Besides protein clustering, an unexpected observation is that STxB binding to a DOPC/sphingomyelin/cholesterol/Gb_3_ (40∶35∶20∶5) lipid mixture results in a redistribution of the fluorophore perylene from the *l*_o_- in the *l*_d_-phase. In GUV experiments, perylene was preferentially found in *l*_o_-phases in ternary lipid mixtures containing *egg* sphingomyelin, DOPC and cholesterol [20]. However, Baumgart et al. [35] reported that perylene does not show any favored localization in ternary mixtures containing *brain* sphingomyelin. Even though we used *brain* sphingomyelin, perylene is unambiguously localized in the *l*_o_-phase in solid supported membranes composed of sphingomyelin, DOPC and cholesterol before the addition of protein. After protein binding, the fluorophore is colocalized with TexasRed DHPE in the *l*_d_-phase. Following the idea that the protein generates a new membrane organization comprising STxB-Gb_3_ complexes that are tightly packed, the fluorophore is excluded from this further compressed membrane phase and thus partitions into the *l*_d_-phase. This notion is in agreement with the increased height difference observed in the topography images shown in Figure 2 and 3 and the fact that the total area occupied by *l*_o_-phase is reduced by 10% upon protein binding. In addition, the circularity of the *l*_o_-domains increases after protein addition, which might be a result of an increased line tension owing to the increased height difference between the *l*_o_-domains and the surrounding *l*_d_-phase membrane. Recently, Garcia-Sáez et al. [36] also showed that an increase in line tension induced by a hydrophobic mismatch in solid supported membranes leads to an increase in circularity of the membrane domains. We thus conclude that a lipid compaction in the *l*_o_-phase occurs, which is induced by the bound protein. We suggest that Gb_3_ lipids compact upon STxB binding, leading to lipid reorganization within the membrane and eventually results in the formation of an additional membrane phase enriched in Gb_3_ and STxB. Even though the lipid organization and the composition of this new phase is not yet fully understood, it is well conceivable that the packing parameter of Gb_3_ might influence this lipid compaction and reorganization process, which was corroborated by fluorescence overlay experiments. Initially phase separated planar membranes containing Gb_3_ with a long chain saturated fatty acid (22∶0) demix in the presence of STxB similarly to what has been observed for membranes containing porcine Gb_3_, which is composed of 70% saturated and 30% unsaturated fatty acids. In contrast, STxB is rather homogeneously bound to membranes containing Gb_3_ with a mono-unsaturated fatty acid (22∶1^13^), which is less densely packed, and is not organized in macroscopic domains, from which perylene is excluded.

The proposed lipid compaction was further supported by monolayer experiments at the air/water interface, demonstrating that new *l*_o_-phase domains are formed upon STxB binding and a denser lipid packing is induced within already existing liquid ordered phase domains. That the mutant W34A-STxB does not influence the phase behavior of the monolayer further suggests that Gb_3_ clustering underneath STxB is presumably a prerequisite for the formation of *l*_o_-phase domains.

**Figure 9:**
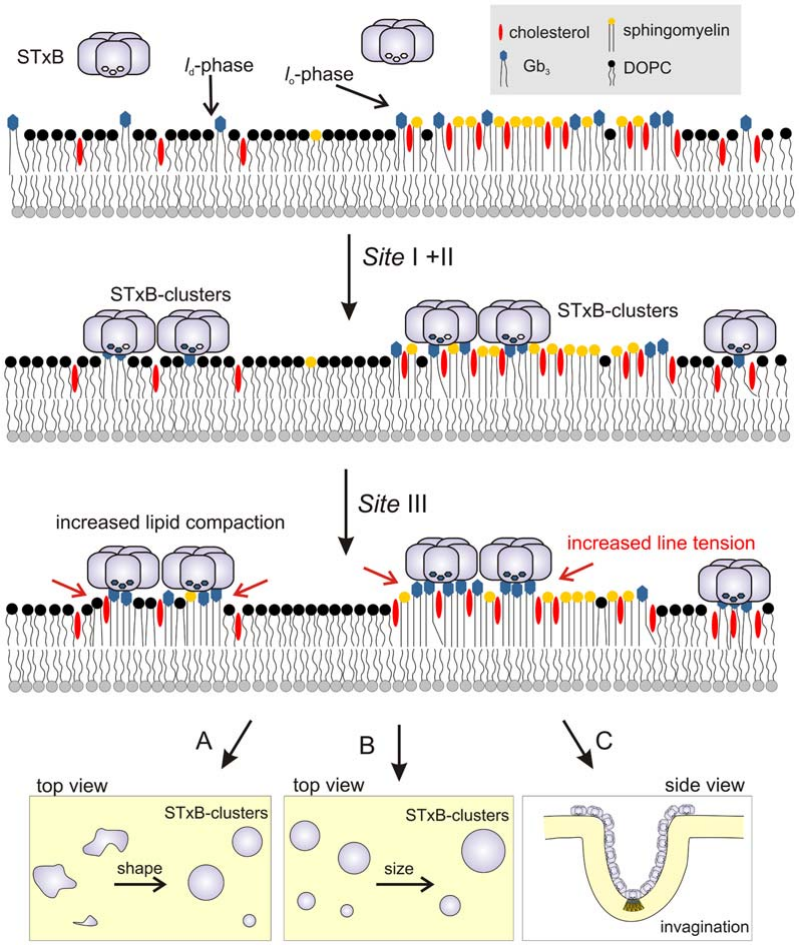
Model of the binding modes of STxB to Gb_3_ containing membranes. The organization of the lipids in the bottom leaflet of the bilayer is not given in detail. For further details see text.

Based on our current knowledge, we propose a model for protein binding and lipid reorganization as depicted in Figure 9. First, STxB binds via the high affinity binding sites I and II to Gb_3_ and forms protein clusters. Via binding site III, more Gb_3_ molecules are recruited underneath the protein, which results in membrane thickening and a close packing of the lipids. As a result of the increased line tension (red arrows) the membrane domains become more circular (A) and fuse to larger domains (B) in case of planar supported membranes [9], [37], [38]. In case of free membranes, such lipid reorganization and compaction generated by Gb_3_ clustering can result either in large membrane domains with the formation of only small buds (see Figure 1B) or, if no large phase separation occurs, it leads to bending of the membrane (C) if the membrane tension is low enough (see Figure 1A). In conclusion, the presented results provide evidence for the STxB-induced formation of tightly packed membrane domains, whose intrinsic properties such as height mismatch and trans-bilayer stress may contribute to the driving forces required for the clathrin-independent plasma membrane invaginations in cells.

## Materials and Methods

### Materials

1,2-Dioleoyl-*sn*-glycero-3-phosphocholine (DOPC) was purchased from Avanti Polar Lipids (Alabaster, AL, USA); sphingomyelin, cholesterol, and the fluorescently labeled lipids Sulforhodamine 101 DHPE (TexasRed DHPE) and perylene were from Sigma-Aldrich (Taufkirchen, Germany); porcine globotriaosylceramide (Gb_3_) was from Matreya (Pleasant Gap, PA, USA), Gb_3_ (22∶0) and Gb_3_ (22∶1^13^) was synthesized according to the procedure described previously (9). *β*-BODIPY 500/510 C_12_-HPC (Bodipy-PC) was obtained from Molecular Probes (Eugene, OR, USA). Recombinantly expressed STxB and W34A-STxB as well as the modified Cy3-labeled STxB was purified as previously described [39]. All protein concentrations are referred to the molar mass of the STxB pentamer. Cantilevers (Ultrasharp CSC38) were purchased from Mikromasch (Tallinn, Estonia). Ultra-pure water (specific resistance of 18.2 MΩ/cm) was used, obtained from a Milli-Q purification system (Millipore, Billerica, MA) consisting of a Milli-RO3 plus and a Milli-Q plus 185 ion exchanger.

### Giant unilamellar vesicles

GUVs were prepared by the electroformation technique as previously described [9], [40]. Lipids were dissolved in the appropriate ratio in chloroform at a total concentration of 0.5 mg ml^−1^. GUVs with a typical diameter of 10–30 µm were grown in a sucrose solution adjusted to 300 mOsm. They were transferred into a chamber containing Cy3-labeled STxB (200 nM) in PBS buffer.

### Solid supported bilayers

Bilayers were prepared on mica surfaces by means of vesicle spreading. Stock solutions of DOPC, sphingomyelin, cholesterol, TexasRed DHPE, and Bodipy-PC, respectively were prepared in chloroform, while Gb_3_ was dissolved in chloroform/methanol (2∶1). Lipid films were formed at the bottom of glass test tubes under a stream of nitrogen at *T* = 55°C, desiccated under vacuum at 55°C for 3 h and stored at 4°C. Multilamellar vesicles (MLVs) were obtained by allowing the lipid films to swell in buffer solution (20 mM TRIS/HCl, 100 mM NaCl, 1 mM CaCl_2_, pH 7.4) for 20 min at 55°C followed by vortexing three times for 30 sec every 5 min (total lipid concentration: 0.3 mg ml^−1^). MLVs were subsequently converted into large unilamellar vesicles (LUVs) by extrusion using a mini extruder (LiposoFast, Avestin, Ottawa, Canada) supplied with a 50 nm polycarbonate membrane at 55°C. The LUVs were deposited on a freshly cleaved mica plate fixed in an open Teflon chamber with a lipid concentration of 0.3 mg ml^−1^ in the presence of 10 mM Ca^2+^. After 1 h incubation at 55°C, the mica surface was rinsed with PBS buffer (1.5 mM KH_2_PO_4_, 8.1 mM Na_2_HPO_4_, 2.7 mM KCl, 136.9 mM NaCl, pH 7.4) at room temperature. Either Cy3-labeled or unlabeled STxB was added to the planar bilayer at room temperature. Non- and reversibly bound protein was removed after 1 hour by rinsing with PBS buffer.

### Lipid monolayers at the air/water interface

STxB interaction with lipid monolayers at the air/water interface was investigated by means of a film balance equipped with a Wilhelmy system (Riegler & Kirstein, Potsdam, Germany). To reduce the volume of the subphase after monolayer compression, a small trough with a total volume of about 6 ml was placed into the Teflon trough of the film balance. Lipid mixtures dissolved in chloroform/methanol were spread onto a PBS buffered subphase and the lipid film was equilibrated for 15 min. After the monolayer was compressed with a velocity of 4.7 mm^2^ s^−1^ to a surface pressure of 30 mN m^−1^, the total subphase volume was reduced by delimiting the inserted small trough with two Teflon pieces and the film was equilibrated for another 15 min. STxB with a final concentration of 60 nM was injected into the subphase without disturbance of the monolayer via an inlet port of the small trough. The lateral organization of the lipid monolayer doped with 1 mol% Bodipy-PC and the localization of Cy3-labeled STxB was investigated by fluorescence microscopy, while the surface pressure was monitored simultaneously. All measurements were performed at *T* = 20°C.

### Fluorescence microscopy

Fluorescence images of planar lipid monolayers and bilayers were obtained with an Axiotech Vario microscope (Carl Zeiss, Jena, Germany) equipped with an EC Epiplan-Neofluar 20×/0.5 objective (Zeiss) and filter set 44 (BP 475/40, FT 500, BP 530/50, Zeiss) for monolayer inspection and a water immersion objective Achroplan 40×/0.8 W (Zeiss) for fluorescence analysis of solid supported bilayers. Filter set 45 (BP 560/40, FT585, BP 630/75, Zeiss) was used for Cy3-STxB and TexasRed DHPE fluorescence light detection and filter set 47 (BP 436/20, FT 455, BP 480/40, Zeiss) for perylene fluorescence.

Fluorescence images of GUVs were obtained at room temperature with a confocal microscope (LSM 510, Carl Zeiss, Jena, Germany) equipped with an oil immersion objective 63× PL APO HCX, 1.4 numerical aperture (Zeiss).

### Scanning force microscopy

Surface images of solid supported bilayers were obtained in an open Teflon fluid chamber using a JPK NanoWizard II scanning force microscope (JPK Instruments, Berlin, Germany). Measurements were performed in PBS buffer in contact mode using microfabricated silicon tips with a typical resonance frequency of 10 kHz and a nominal spring constant of 0.03 N m^−1^. Scan speed was adapted to the scan size and set between 0.6 and 2 Hz. Image resolution was 512×512 pixels. Height differences were extracted by pixel analysis of several SFM images with different image sizes.

## Supporting Information

Figure S1 Solid supported bilayer composed of DOPC/cholesterol/porcine Gb_3_ (65∶30∶5) in the absence of protein. The topographic SFM image of the bilayer shows no phase separation. Scale bar: 1 µm.
(0.40 MB DOC)

Figure S2 Typical histogram of the height difference analysis between STxB and the *l_o_*-phase of solid supported bilayers composed of DOPC/sphingomyelin/cholesterol/Gb_3_ (40∶35∶20∶5). The histogram was obtained from areas of the SFM images, where only STxB bound to the *l_o_*-phase was found. The height was determined to (2.2±0.2) nm (*n* = 26).
(0.08 MB DOC)

Figure S3 A Compression isotherms of DOPC/sphingomyelin/cholesterol/porcine Gb_3_ (40/35/20/5) with (red) and without (black) Bodipy-PC on a PBS-buffered subphase at 20°C. The area per molecule is given as an average value of the lipid mixture (*n* = 5), normalized to an *A_20_*_ mN/m_ value of 50 Å^2^. B Compression isotherms of DOPC/sphingomyelin/cholesterol/porcine Gb_3_ (65/10/20/5) with (red) and without (black) Bodipy-PC on a PBS-buffered subphase at 20°C. The area per molecule is given as an average value of the lipid mixture (*n = 5*), normalized to an *A_20_*_ mN/m_ value of 56 Å^2^. For both mixtures, DOPC was replaced by Bodipy-PC.
(0.57 MB DOC)
