# Supplementary material for: Lipid Reorganization Induced by Shiga Toxin Clustering on Planar Membranes
Source: PLoS One. 2009 Jul 16;4(7):e6238. doi: 10.1371/journal.pone.0006238 (PMC2705791; doi:10.1371/journal.pone.0006238)
Supplement: Figure S1 — Solid supported bilayer composed of DOPC/cholesterol/porcine Gb3 (65∶30∶5) in the absence of protein. The topographic SFM image of the bilayer shows no phase separation. Scale bar: 1 µm. (0.40 MB DOC) [file pone.0006238.s001.doc]

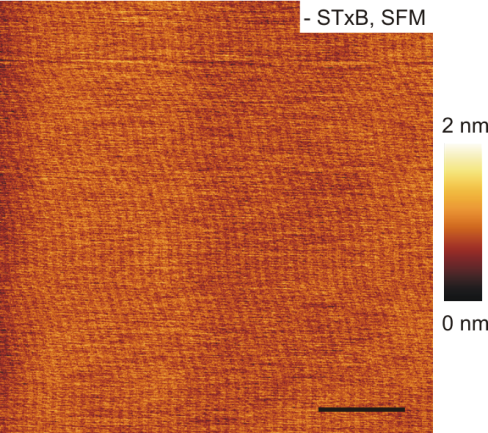


**Figure S1.** Solid supported bilayer composed of DOPC/cholesterol/porcine Gb_3_ (65:30:5) in the absence of protein. The topographic SFM image of the bilayer shows no phase separation. Scale bar: 1 µm.
