# Supplementary material for: Lipid Reorganization Induced by Shiga Toxin Clustering on Planar Membranes
Source: PLoS One. 2009 Jul 16;4(7):e6238. doi: 10.1371/journal.pone.0006238 (PMC2705791; doi:10.1371/journal.pone.0006238)
Supplement: Figure S2 — Typical histogram of the height difference analysis between STxB and the lo-phase of solid supported bilayers composed of DOPC/sphingomyelin/cholesterol/Gb3 (40∶35∶20∶5). The histogram was obtained from areas of the SFM images, where only STxB bound to the lo-phase was found. The height was determined to (2.2±0.2) nm (n = 26). (0.08 MB DOC) [file pone.0006238.s002.doc]

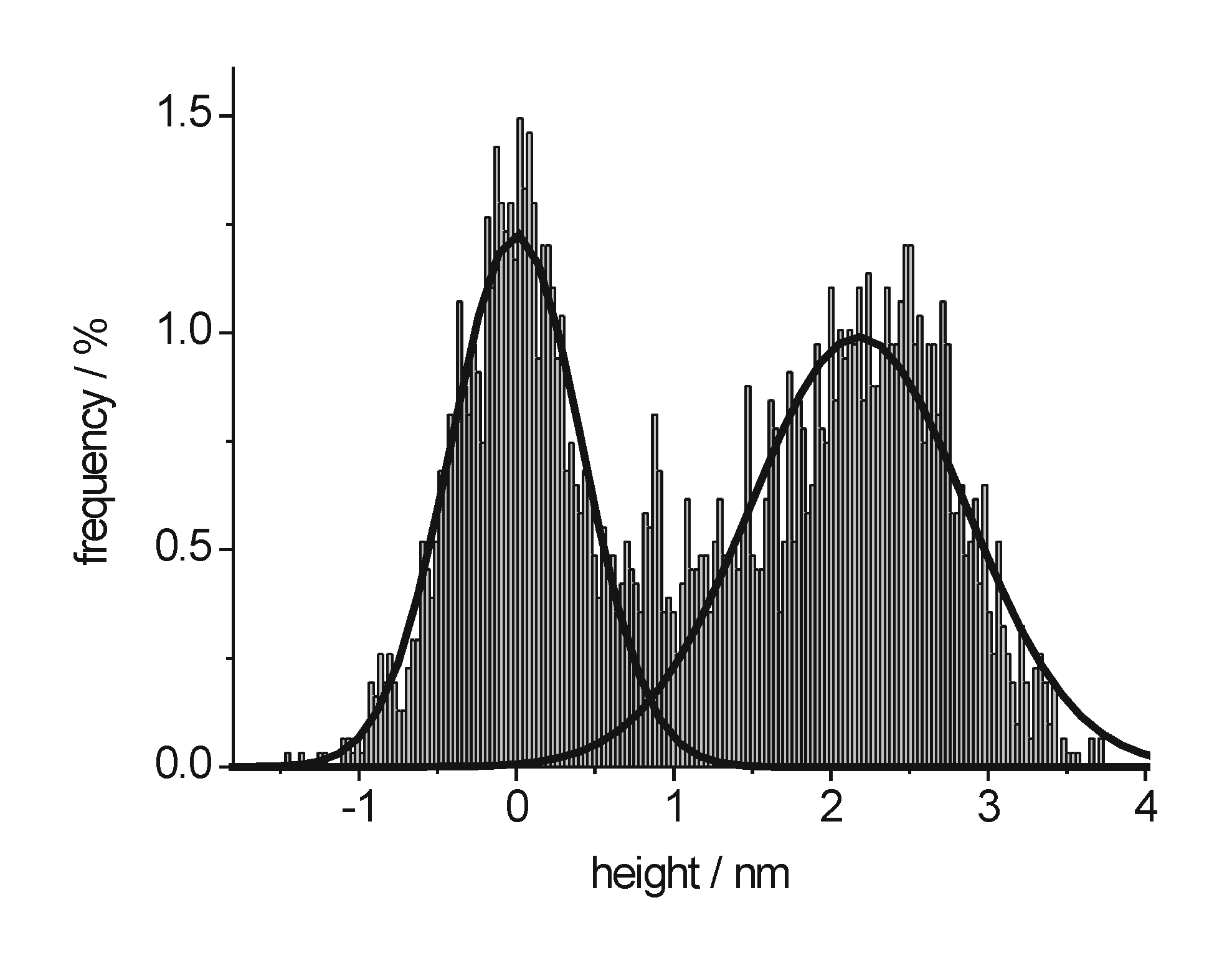


**Figure S2.** Typical histogram of the height difference analysis between STxB and the *l*_o_-phase of solid supported bilayers composed of DOPC/sphingomyelin/cholesterol/Gb_3_ (40:35:20:5). The histogram was obtained from areas of the SFM images, where only STxB bound to the *l*_o_-phase was found. The height was determined to (2.2 ± 0.2) nm (*n* = 26).
