# Supplementary material for: Lipid Reorganization Induced by Shiga Toxin Clustering on Planar Membranes
Source: PLoS One. 2009 Jul 16;4(7):e6238. doi: 10.1371/journal.pone.0006238 (PMC2705791; doi:10.1371/journal.pone.0006238)
Supplement: Figure S3 — A Compression isotherms of DOPC/sphingomyelin/cholesterol/porcine Gb3 (40/35/20/5) with (red) and without (black) Bodipy-PC on a PBS-buffered subphase at 20°C. The area per molecule is given as an average value of the lipid mixture (n = 5), normalized to an A20 mN/m value of 50 Å2. B Compression isotherms of DOPC/sphingomyelin/cholesterol/porcine Gb3 (65/10/20/5) with (red) and without (black) Bodipy-PC on a PBS-buffered subphase at 20°C. The area per molecule is given as an average value of the lipid mixture (n = 5), normalized to an A20 mN/m value of 56 Å2. For both mixtures, DOPC was replaced by Bodipy-PC. (0.57 MB DOC) [file pone.0006238.s003.doc]

**Figure S3.** **A** Compression isotherms of DOPC/sphingomyelin/cholesterol/porcine Gb_3_ (40/35/20/5) with (red) and without (black) Bodipy-PC on a PBS-buffered subphase at 20 °C. The area per molecule is given as an average value of the lipid mixture (*n* = 5), normalized to an *A*_20 mN/m_ value of 50 Å^2^. **B** Compression isotherms of DOPC/sphingomyelin/cholesterol/porcine Gb_3_ (65/10/20/5) with (red) and without (black) Bodipy-PC on a PBS-buffered subphase at 20 °C. The area per molecule is given as an average value of the lipid mixture (*n* = 5), normalized to an *A*_20 mN/m_ value of 56 Å^2^. For both mixtures, DOPC was replaced by Bodipy-PC.
